# Trends in Snapshot Spectral Imaging: Systems, Processing, and Quality

**DOI:** 10.3390/s25030675

**Published:** 2025-01-23

**Authors:** Jean-Baptiste Thomas, Pierre-Jean Lapray, Steven Le Moan

**Affiliations:** 1 Imagerie et Vision Artificielle (ImViA) Laboratory, Department Informatique, Electronique, Mécanique (IEM), Université de Bourgogne Europe, 21000 Dijon, France; 2 Department of Computer Science, NTNU—Norwegian University of Science and Technology, 2815 Gjøvik, Norway; steven.lemoan@ntnu.no; 3 The Institute for Research in Computer Science, Mathematics, Automation and Signal, Université de Haute-Alsace, IRIMAS UR 7499, 68100 Mulhouse, France; pierre-jean.lapray@uha.fr

**Keywords:** spectral imaging, snapshot spectral imaging, image reconstruction, image quality

## Abstract

Recent advances in spectral imaging have enabled snapshot acquisition, as a means to mitigate the impracticalities of spectral imaging, e.g., expert operators and cumbersome hardware. Snapshot spectral imaging, e.g., in technologies like spectral filter arrays, has also enabled higher temporal resolution at the expense of the spatio-spectral resolution, allowing for the observation of temporal events. Designing, realising, and deploying such technologies is yet challenging, particularly due to the lack of clear, user-meaningful quality criteria across diverse applications, sensor types, and workflows. Key research gaps include optimising raw image processing from snapshot spectral imagers and assessing spectral image and video quality in ways valuable to end-users, manufacturers, and developers. This paper identifies several challenges and current opportunities. It proposes considering them jointly and suggests creating a new unified snapshot spectral imaging paradigm that would combine new systems and standards, new algorithms, new cost functions, and quality indices.

## 1. Introduction

Imaging science has enabled scientific progress in countless scientific fields, from space exploration to environmental monitoring, medicine, and conservation science. Spectral imaging (SI), a specific type of imaging technology, combines spectroscopy and imaging to reveal detailed material and compositional information about objects (e.g., a distant planet, a fruit, human skin, etc.) based on how they interact with light in specific wavelength ranges [1]. In 2022, the optical instrumentation and measurement systems market reached a value of USD 16 billion, exhibiting an annual growth rate of 5.5%. Within this market, the sub-segment “spectrometers and spectral cameras” constitutes a significant portion, representing a value of USD 6.5 billion [2] (source provided by Tematys/Photonics21, 2023). In another source, the global spectral imaging market was valued at approximately USD 16.1 billion in 2022 and is projected to reach USD 47.3 billion by 2032, reflecting a compound annual growth rate of 11.3% [3]. In Europe, this number is projected to be 8.47% [4]. Several manufacturers and models of spectral cameras are on the market, ranging from USD 5.000 to USD 200.000 in price, such as Silios Technologies (TOUCAN Cam), Hypsec (VNIR-1800 hyperspectral camera), Spectral Devices Inc. (MSC2-AGRI-1-A camera), Imec (SNAPSCAN VNIR camera), Specim (Specim IQ camera), Sony (IMX454LXR-C), and MICASENSE (REDEDGE-MX), among others.

Spectral imaging has successfully contributed to the development of applications in many fields. General reviews [1,5,6], along with application-specific surveys, such as in agriculture [7], photography [8,9], materials science [10,11], and biomedical imaging [12,13], demonstrate its versatility. SI also has applications in space exploration [14], waste management [15], cultural heritage [16,17], and environmental monitoring [18]. However, several practical limitations to the use of spectral imaging have been found, especially in the biomedical fields [19], where the low spatial resolution of spectral imaging systems can make the analysis of small biological structures difficult, such as for mass spectrometry imaging on tissues [20].

**Figure 1:**
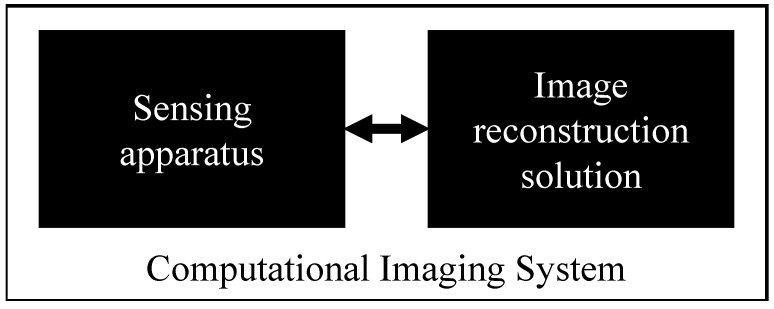
A computational imaging system is composed of a sensing apparatus (hardware) coupled with a computational module (software) that reconstructs a full-resolution image, from limited sampled data captured by the sensing apparatus.

Snapshot spectral imaging (SSI) [21,22] refers to the ability of a spectral imaging system to capture information in only one shot, as opposed to sequential or scanning devices. SSI started to develop in the early 2000s from the need to have more compact and easy-to-use devices in order to enable the development of higher temporal sampling. Indeed, in many applications, e.g., robotics, where spectral imaging can help substantially with automatic scene analysis, rapid feedback is essential, which can only be obtained with SSI. The SSI market held the largest share of the spectral imaging market in 2023 [23] and is expected to grow at a significant rate owing to increasing demand for high-resolution and real-time spectral data. Several SSI technologies exist today [21], with an increasing interest from various application domains for new developments. The field of computational imaging, in particular, has seen the emergence of a new community of researchers and end-users focused on super-resolution, interpolation, and enhancement methods to reconstruct full-resolution images or videos from low-resolution sensors data [24,25], considered to be raw data (see Figure 1).

**Figure 2:**
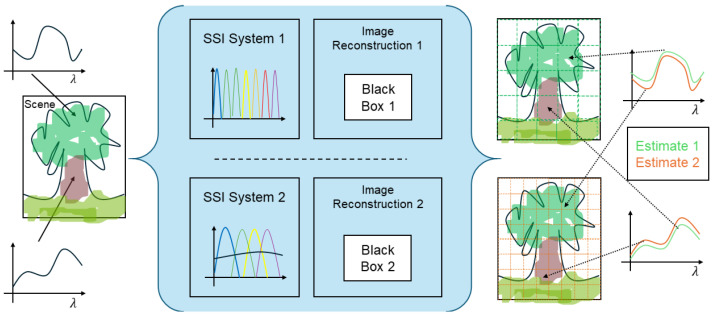
A scene with two materials of specific reflectance functions (**left**). Two different users may possess different systems (**middle**) and measure different information, which may mislead their analysis (**right**).

SSI systems may be designed in a variety of ways [21,26], but typically involve low-resolution sensing that is augmented by some kind of image reconstruction method (e.g., demosaicing, and/or super-resolution) and calibration to recover the useful spatio-spectro-temporal resolution as well as the dynamic range. Due to the variety of sensing apparatus and their specific individual image reconstruction solutions, and the lack of open-source standard benchmark datasets and quality assessment methods, two SSI devices developed independently may lead to significantly different results [27], as shown in Figure 2. This observation is crucial in understanding the motivation and the trends for future development. One scene captured by two different SSI systems will provide information of different quality. This is illustrated in Figure 2, where a measured spectrum from one system can be significantly different from another. This can potentially hinder the extraction of meaningful information from the data and make interpretation inconsistent. Today, users have no way to assess the quality of their measurements, nor any standards to refer to.

This challenge is not new in imaging science. For example, in colour imaging, each camera model (e.g., from one manufacturer to another) captures potentially different spectral bands and uses different proprietary image-processing software. Despite this diversity, standardised colour spaces based on colour science principles are now well-established, along with colour management frameworks and device characterization data (such as ICC profiles following the International Color Consortium format) that ensure consistent colour representation across devices. Additionally, a rich tradition of quality assessment for colour images exists, employing both quantitative metrics and subjective evaluations. Inspired by these developments, we infer that the creation of a unified spectral paradigm, which will facilitate the design and use of SSI by many communities, is needed. By defining effective, stable, compact, and easy-to-use SSI, the research community should provide new measurement tools to a broad range of scientific fields, which will enable advances in our understanding of the physical world. The goal of this article is to survey the different aspects that need to be unified, and to discuss how to construct such a new paradigm. We do not perform an exhaustive survey for all the specific aspects; rather, we focus on the definition and illustration of the major tendencies and promising approaches.

The article is structured as follows. First, a spectral imaging model is presented in Section 2 that mathematically formulates the process of spectral image formation, together with an explanation of the SSI pipeline. Section 3 covers the advances and trends in imaging technology and standardization. Section 4 considers the image reconstruction algorithms, and Section 5 develops the approaches related to image quality. As a conclusion, we discuss the potential unification in the SSI paradigm in Section 6 and its impact on different communities.

## 2. Spectral Imaging

### 2.1. Imaging Model

When light (electromagnetic energy) encounters a material, it can be absorbed, scattered, reflected, or transmitted. The energy that is reflected relative to the incoming energy provides information related to material and surface properties, which is the interest of spectroscopy. The spatial variation of this light–energy interaction (i.e., an image, described as a projection of this energy on a plane) provides insight about the shape and the nature of the object composed of this material, and ultimately allows for scene analysis; hence, there is interest in SI, also referred to as *reflectance spectroscopy imaging*. When we investigate the techniques that allow us to capture digital spectral images, we can identify that we essentially sample the scene across four dimensions, namely the spatial, spectral, temporal, and intensity dimensions of light, in order to measure one digital value related to the quantity of light, per spatial location, for a specific wavelength or set of wavelengths, across a certain time. The polarization sensitivities of spectral systems are neglected in the following imaging model. However, these effects can occur more or less pronouncedly, depending on the capture technology used, which is discussed in Section 4.3.

The irradiance integrated by a camera sensor at the image plane J(λ,x,t) can be represented by Equation (1), as follows:

(1) $$\begin{array}{c} I_{s}\left(X,T\right)=ADC\left(\int_{\Lambda }\int_{X}\int_{T}J\left(\lambda ,x,t\right)s\left(\lambda ,x,t\right)d\lambda dxdt+\sigma \right)\;, \end{array}$$

with J(λ,x,t) being the irradiance reaching the sensor from the radiant material and s(λ,x,t) being the spectral sensitivity of the sensor, both at the position *x* ($x\in R^{2}$) of the image plane, as a part of one pixel *X*, and at the time *t*. The integration is continued for a defined time *T*, referred to as the *integration time* of the pixel (in general, *T* is defined for all the pixels of one sensor). In the case of moving elements in the scene, the irradiance at location *X* varies with time. The spectral sensitivities of the sensor only vary along *t* within a time-scanning technology, e.g., a colour wheel. In practice, the quantity of energy captured, $I_{s}\left(X,T\right)$, is digitised using an analogue-to-digital converter (ADC). It is important to understand that this digitisation needs to be in accordance with the quantity of energy unless you have over-exposed or under-exposed signal, which is a challenge for a sensor that has the same *T* for every pixel. If we consider an additive noise σ, then an under-exposed signal shows a weak signal-to-noise ratio. $I_{s}\left(X,T\right)$ is the raw data captured by the imager; that is the only information accessible by the system, and it is later used to estimate a version of J(λ,x,t) thanks to an imaging pipeline.

To measure the properties of materials, it is relevant to relate the irradiance J(λ,x,t) to optical material properties. In order to do that, it is required to assume a light-interaction model [28] to access, e.g., reflectance factors. A material’s ability to reflect a part of the light is generally an important information and is described by its bidirectional reflectance distribution function (BRDF) [29]. In many cases, a simplification is performed where the material is considered Lambertian. In such a hypothesis, we can additionally write J(λ,x,t)=r(λ,x,t)l(λ,x,t) with r(λ,x,t), the reflectance factor per wavelength in the radiant direction to the camera, which varies with *t* in case of moving objects. Furthermore, l(λ,x,t) is the spectral power distribution of the light illuminating the object, which may also vary with time. Despite being often used, we remind the reader that this never happens in reality and that it is an approximation made to simplify the model.

In this well-used simplified model, everything is considered stable or averaged over the integration time for a given pixel; thus, we obtain Equation (2) for each pixel, *X*, as follows:

(2) $$\begin{array}{c} I_{s}\left(X\right)=ADC\left(\int_{\Lambda }r\left(\lambda \right)l\left(\lambda \right)s\left(\lambda \right)d\lambda +\sigma \right)\;. \end{array}$$

To conclude, $I_{s}\left(X\right)$ is the measurement we make (raw data for the specific spectral sensitivity s(λ)) to estimate $\tilde{J}\left(\lambda ,x,t\right)$, and the challenge for an imaging pipeline is to obtain the best estimation of J(λ,x,t) and related quantities (e.g., r(λ)) by designing performant systems, algorithms, and quality measures. In the following, we refer to I(X) as the complete set of raw data from snapshot capture.

### 2.2. Imaging Pipeline

**Figure 3:**
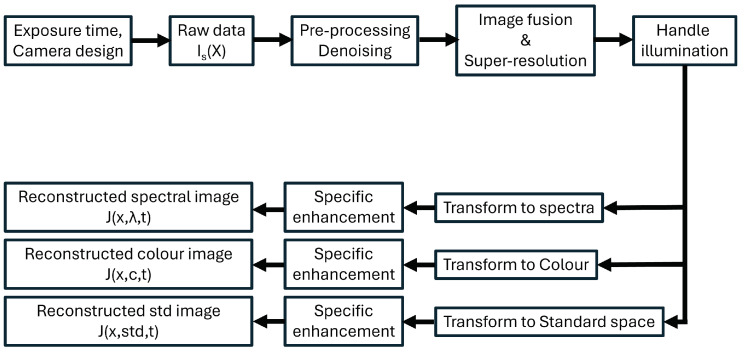
The camera captures raw data, the characteristics of which depend on the technology. Essentially, these data undergo some transformations, which include reconstructing the full image information through the fusion and integration of all the raw data. We consider that the pipeline may be fine-tuned to a specific output, such as a colour image, a radiance or reflectance image, or an image in a specific standard space of any kind. This pipeline is a conceptual example, and often blocks are in different orders or repeated in the literature or in other systems. Furthermore, in specific systems, image fusion and super-resolution have specific names, like demosaicing in spectral filter arrays, or image registration in the case of multiple camera systems.

The imaging pipeline receives the raw data from the sensor system as its input, i.e., I(X), and aims to provide one version of J(x). This is performed by taking into account the nature of I(X), which can vary between systems, and by also taking into account an imaging model, which may be more or less complex. A generic model of an imaging pipeline is shown in Figure 3. Examples of different imaging pipelines can be found in the literature, e.g., [30]. The order of modules may vary from pipeline to pipeline. Each module, set, subset, or combination of modules is optimized according to specific quality criteria, hence resulting in the impact of image quality index. It is noteworthy that speed and energy performance are also very important, especially in the case of portable, wireless devices working on batteries, e.g., smartphones. This implies a limited computational power and usually demonstrates a drop in image quality performance. Furthermore, in many cases, it is important to understand the minimum quality expected by the user. Illumination is an important factor in imaging, and, in spectral imaging, two solutions co-exist, as follows: the standard protocol is a calibration with a white diffuse patch, which implies that the illumination will not change over time; the second solution is a relative calibration based on scene statistics analysis to perform spectral constancy (analogue to computational colour constancy) across changes in light spectral properties [31].

In Figure 3, we also show that, in the case of spectral imaging, several output are possible. The straightforward output is an image reconstructed according to a standard, which could be either the native spectral bands of the camera (like in the case of many snapshot camera systems, or remote sensing optical sensors such as Landsat-8). Other types of standards can also be considered. In several cases, such as laboratory-calibrated setups, users are interested in working with an estimation of spectral radiance or reflectance factors. In the case of colour imaging [32], a colour image according to an RGB standard is often desired by the users, or sometimes a colorimetric image is required. It is possible and often useful to include an image enhancement module to improve the image specifically of the output, which could comprise denoising, smoothing, contrast boosting, etc.; these could be applied to all or part of the image, locally or globally.

**Figure 4:**
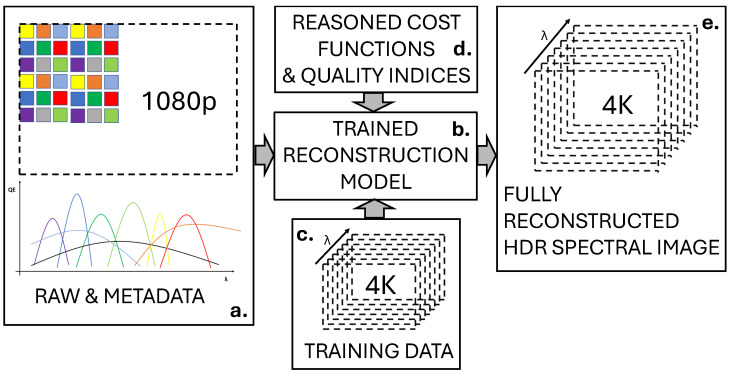
Summary of an image reconstruction algorithm for SFA raw images. From the raw image and its associated metadata, such as spectral sensitivities and filter arrangement (**a**), the reconstruction model (**b**) is trained on existing data, which are often full resolution spectral images from which raw data are simulated (**c**), and cost functions (**d**), in order to reconstruct the image in all the required dimensions (**e**).

To provide an example, a typical setup is shown in Figure 4 for the technique known as spectral filter arrays (SFA) [26]. A filter array can be defined by its superpixel (or moxel or mosaic element), which corresponds to a predefined pattern composed of a set of geometrically arranged spectral filters. This pattern is repeated over the entire surface that makes up the focal plane of the sensor. This has specific features that define I(X). An important aspect of the imaging pipeline in such an example is reconstructing the spatial dimension, using a specific algorithm named demosaicing. In this specific example, the spectral resolution and HDR (high dynamic range) content are also jointly estimated from I(X). The related algorithm can be trained and validated on a specific dataset, according to specific quality indices, which gives the reconstructed image $\tilde{J}\left(\lambda ,x\right)$. For another type of technology, the sampling is performed differently, and so the reconstruction is different.

## 3. System and Standardisation

Generally, the objective of a spectral camera system is to provide the best possible quality of I(X) so that $\tilde{J}\left(\lambda ,x\right)$ can be accurately estimated later.

Spectral selection has historically been achieved sequentially using a filter wheel combined with optical bandpass filters [33], where the camera system produces one channel image per set of wavelengths. Several technologies have been developed [26], such as those based on liquid crystal tunable filters [34] or tunable illumination [35]. Today, the most well-known systems are the pushbroom systems, which sample spatio-spectral domains over time. The disadvantage of these systems, besides their mechanical complexity and costs, is their inability to capture moving objects in a video stream at a reasonable frame rate. Thus, they cannot aid in the observation of dynamic events or mechanisms, while many scientific fields would benefit from the ability to observe the evolution of rapid phenomena from spectral images.

**Snapshot spectral imaging** (SSI) refers then to the ability of a spectral imaging system to capture information in only one shot. It is thus essentially a sampling process where the light intensity, the spatial resolution, and the spectral resolution are limited to allow for one single take. SSI relies on several techniques to capture more or less well-sampled dimensions. The sampled data are then used to estimate the captured image values (e.g., [36]), as covered in Section 4. Usually, these reconstruction techniques are driven by cost functions related to the quality of the estimation, which relates to the quality of images; this is discussed in Section 5.

As mentioned in the Introduction, several commercial systems exist on the market, including SSI options. However, none of the products allow for the traceability of errors in the estimation of the measured quantities, e.g., irradiance J(λ,x,t) or reflectance factors r(λ,x), which may mislead the people using the data; also, none of the solutions were designed as a whole in a transparent manner. In fact, it was demonstrated that there were great variations in the quality of data captured by spectral imaging systems used by different groups for cultural heritage applications [37,38]. This is the result of a combination of different acquisition protocols and conditions, systems, algorithms, and quality evaluations at all levels. Similar variations also occur in the spatial, temporal, and intensity domains.

### 3.1. Technologies for SSI

Many efforts have been initiated to develop SSI over the last few decades. In general, these techniques revolve around the concept of having one or several solid-state image sensors, augmented with a system that permits the sampling of spatio-spectral dimensions, according to the light and the temporal sampling ability of the sensors. The major techniques were defined 10 years ago, and no major conceptual breakthroughs have happened since the review by Hagen and Kudenov [21]. However, many demonstrators have been implemented and reported in the literature. A major current advancement related to the development of sub-wavelength optics to create metasurfaces and three-dimensional (3D) metaforms to filter the light. Hereafter, we review the major SSI techniques.

- Multispectral beamsplitting: It employs a combination of beamsplitters and several sensors to separate light components [39]. This is an extension of the colour broadcasting camera, which enables high-resolution imaging [40]. However, the higher the number of prisms, the more the noise and the price increase; eventually, commercial systems for spectral imaging limited the number of sensors to three. However, several commercial systems are available (e.g., [41]). This technique is particularly well suited to cases where several sensors need to be combined to capture a large interval of the electromagnetic spectrum, i.e., visible and near-infrared (VNIR) or short-wave infrared (SWIR).
- Coded aperture snapshot spectral imager (CASSI) [42]: It is predicated on the principles of compressive sensing theory, which enables signal reconstruction for sampling rates below the Nyquist frequency. This technique attracted interest in the signal-processing community, and many scientific publications relate to this technique (e.g., [43,44]), but the physical realisation of the coded aperture mask together with the processing module is challenging [45]. Generally speaking, the advantages of CASSI include its sensitivity, rapidity, and small data [46]. The challenges of CASSI are related to image reconstruction complexity and the design of the pattern.
- Integral field spectroscopy: It encompasses the lenslet array methodology [47,48]. Video-rates sensors have been researched since 2009 [49,50], have recently been followed by regular and current developments [51]. The most recent advances include the use of filters based on metasurfaces [52].
- SFA technology [26,53,54]: It employs a filter array to sample the spectral dimension. It attracted great interest in the imaging community since its concept is very close to that of a colour filter array (CFA) (see Figure 4). In fact, this technique is a generalisation of its colour pendant that trades spatial resolution for spectral resolution. The traded spectral resolution is usually compensated for by a demosaicing algorithm. Its limitation, specifically its resolution, has been addressed through the fusion of a colour image with an SFA image [55]. The current evolution of SFA is related to improvements in the filtering process. We can select filters based on nano-technologies. The nanometric control of matter would allow for several opportunities, such as nanowires for the better control of bandpass [56]. In addition, reconfigurable filters [57] would allow for an increase in spectral resolution but would come at the expense of the temporal dimension.

Other techniques are relevant but were mostly not developed to an end-user commercial level. There are several factors for this; it is either due to cumbersome or expensive optical features, or sometimes due to the low level of maturity of the technology. Other times, the general performance was not at the level of other techniques, but this could change in the future. We list several of these other technologies below:

- Computed tomography imaging spectrometry [58,59]: employs computed tomography;
- Tunable echelle imager [60]: employs tunable echelle gratings;
- Image-replicating imaging spectrometer [61,62]: employs image replication methodologies;
- Image mapping spectrometry [63,64,65]: correlates images with spectral data;
- Snapshot hyperspectral imaging Fourier transform spectrometer [66,67,68,69,70,71]: employs Fourier transform techniques;
- Multispectral Sagnac interferometer [72]: employs Sagnac interferometry;
- Vertically stacked photodiodes [73,74]: employs vertically stacked photodiodes for spectral imaging.

**Figure 5:**
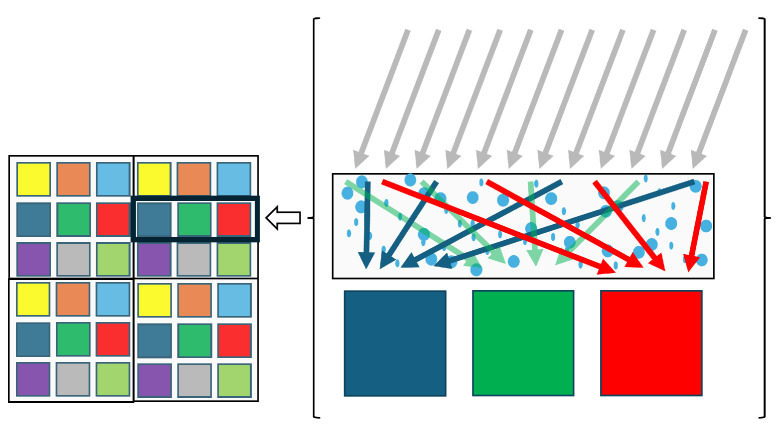
On the left, pixels from the sensor are covered by the metamaterial (e.g., photonic cristal). On the right, two-dimensional (2D) rotated cuts provide an example on how the incoming light reaching the filters is redirected toward each of the sensor’s pixels. Please note that there is no or very little rejection or loss of light, and that nearly all the incident light is exploited.

The major fundamental (r)evolution in SSI is the emergence of spectral routers [75]; indeed, by combining the concepts from sub-wavelength optics and free-form optics, it is possible to implement 3D metaform-based spectral routers [76]. These can be fabricated using, e.g., the two-photon lithography technique [77], which enables the 3D nanofabrication of transparent patterns with a subwavelength resolution (<200 nm) [78,79]. Numerical optimization based on inverse design [80,81] can then be used to design different families of spectral routers based on periodic, binarized, or a combination of both subwavelength structures [82]. The best design can be decided according to quality indices and algorithm performance [83]. A conceptual example of the use of spectral routers is shown in Figure 5; specifically, the case of a hybrid system between lenslet and a filter array. In Figure 5, the spatial resolution is defined by the block of 3×3 sensor pixels; each of these cells acts as a light collector which has energy at certain wavelengths only, which are related to the sensor sensitivity. This technique would drastically increase the efficiency of the spectral filtering process, allowing for faster imaging and reduced noise. In particular, a strong reduction of cross-talk is reported in the literature. Spectral routers were demonstrated for RGB [80,84,85,86] and RGB-NIR [81,87]. The major asset of such an approach is that ideally no light is lost through the rejection filters, but all the energy is used, which solves one major issue of SSI; that is, the little quantity of light passing through each spectral filter, i.e., the term s(λ) in Equation (2). It is expected that new generations of filters will rise and that SSI will make progress thanks to this technique.

### 3.2. Standardisation and Calibration

Since SSI is an emerging technology, there is a lack of standardization and a need for adequate tools to characterize and qualify systems.

#### 3.2.1. Data Representation Space

**Figure 6:**
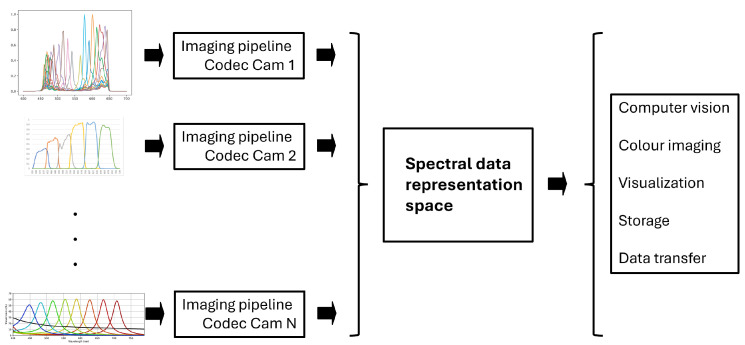
N cameras have N different sets of sensitivities (**left**). It is necessary to create a standard to represent the data from all of these sensors (**middle**), so they can be used independently of the technology (**right**).

The need for a standardized data representation space for snapshot spectral imaging was explained by Thomas et al. [27]. In fact, in the case of hyperspectral imaging, the de facto space is radiance factors or reflectance factors. In the case of a colorimetric system, tristimulus values are estimated or computed. In the case of a colour imaging, the RGB values of specific features are computed. In the case of snapshot imaging, firstly, the time constraints impose the use of a more compact representation than data at a wavelength resolution. Secondly, the diversity of systems require a common representation space (see Figure 6), which could be similar conceptually to an RGB space in the colour domain or which could be a space where information is more compact than in the domain of wavelengths.

This issue also has a major impact on the portability of machine learning solutions trained on data from one camera and used on data from another [88]. The impact of different dimensional reduction on applications was also demonstrated [89]. Similar issues are reported in the remote sensing research community where it is challenging to transform data from, e.g., Landsat-8 to Sentinel-2 [90].

Toward this goal, the pioneer work is from Nambu et al. [91], who proposed a virtual sensor based on Gaussian sensitivities spanning the visible domain. This was followed by Derhak and Rosen [92] with the LABPQR proposal. Then, Thomas et al. [27] proposed an alternative to Nambu et al., using sensitivities inspired by the visual system rather than Gaussian curves as a virtual sensor. This topic is currently being discussed extensively at the Spectral Imaging Research Forum [93] at the division 8, Image Technology, of the Commission Internationale de l’Eclairage, CIE. To our knowledge, there are several directions currently under consideration, as follows: (1) select a few semantically meaningful bands; (2) select effective regular bands that could be Gaussian-like sensitivities; or (3) use dimension reduction techniques on a specific dataset to identify free-form bands that would display the best performances. Among these, there are linear methods and non-linear methods, and also reversible and non-reversible methods. The topic is closely related to the quality of spectral images. It is also closely related to security and encryption in several applications. Major developments are expected to happen in the next years. It is also notable that there is already a Technical Report from the CIE that considers the spectral image format [94].

#### 3.2.2. Calibration

Imaging systems calibration uses imaging test charts usually associated and related to standards, e.g., ISO-12233 “Digital Resolution Still Camera Test Chart” relates to ISO 12233:2024 “Digital cameras—Resolution and spatial frequency responses” [95] or ISO-14524 “Digital Camera Contrast Chart” relates to ISO 14524:2009 “Photography—Electronic still-picture cameras—Methods for measuring opto-electronic conversion functions (OECFs)” [96]. The case of spectral cameras has very limited similar supports. There is an initiative with the IEEE P4001 [97] to characterize spectral imagers. The European Machine Vision Association, EMVA [98,99] has developed a standard for machine vision cameras, but it is not directly usable for SSI. Nothing, to our knowledge, is defined specifically for SSI.

It is, however, important that people developing SSI create such initiatives and carry out the following steps:

- Define the required updates in terminology:
  – Metrological terms must be used in an appropriate manner, e.g., *reflectance factors* must be used over the term *reflectance* in general.
  – Specific items, bands, conversion matrices, etc. must be defined.
  – Spectral camera characterization methods (i.e., the relationship between raw data and radiance or reflectance factors) must be beyond the peak sensitivities of the camera.
- Define metadata structures for SSI:
  – The conversion accuracies of the methods used to transfer raw data to spectral radiance or reflectance factors.
  – Technology specifications need to be included in metadata, e.g., in SFA, spectral sensitivities and spatial arrangements should be included.

These initiatives may also include the design of test charts to qualify SSI systems and reference datasets for quantifying performance, together with quality metrics.

### 3.3. Spatio-Spectral Compromise

The performance and uses of spectral imaging are yet to be investigated and supported by quantitative analysis beyond the current academic publications. It is clear that fusing NIR information to RGB images will enable better performance in several applications than that obtained by only using RGB (cf. Section 1). However, the increase in spectral bands in the visible range can be discussed, in particular in the context of SSI, where spectral information increases with a decrease in spatial resolution.

Porebski et al. [100] compared texture classification from spectral and colour images. They showed that spectral images usually performed the best, whereas colour could perform better in some particular cases. However, in their experiment, the spatial resolution of both setups remained the same. In reality, we trade the spatial resolution for spectral resolution; then, it is not quantified what the results of such a competition, based on imaging systems that exhibit real-world limitations, would be. What is the optimal compromise of spectral and spatial resolution? This question is even more relevant today, since many algorithms reconstruct radiance factors from RGB pixel values with very good performance [101,102,103]. This last approach has some limitations that must be analyzed and quantified [104].

## 4. Image Reconstruction

Among all the spectral imaging technologies described in Section 3, the raw data captured by a system can be sparse, uncalibrated, misaligned, spatially irregularly distributed among channels, noisy, or lack spatial or spectral resolution. This can lead to a misinterpretation of the information by any high-level analysis algorithm. Some technologies will cut more or less in one dimension rather than another. The challenge is to compensate for the optical limitations of systems through digital image processing [22].

### 4.1. Spectral, Spatial, and Intensity Reconstruction

Computer vision algorithms assume that input data (i.e., spectral channels) are perfectly aligned, which is rarely the case in reality, where translational, rotational, and scaling deformations among spectral channel images can occur. Data registration in SSI is based on a high correlation between spectral bands. This might be more or less correct, and this might depend on the scale of observation (high or low frequencies). However, in the context of SSI, the considered range falls within a fairly narrow spectral interval, and adjacent bands usually have a high correlation (except at specific rupture points in the spectra, e.g., at the shift between visible and NIR). Thus, several data registration methods are based either on Fourier transforms [105], local correlation analysis [106], or SIFT descriptors [107], or pansharpening [108,109,110,111]. Moreover, when the number of channels is high, images can suffer from very low spatial resolution. This limits their usability in applications that need to discriminate small details in a scene. Super-resolution techniques are often applied to reconstruct high-resolution images from low-resolution data [112,113], using a single frame [114] or a video sequence [115,116]. In the specific case of filter array sensors, the spatial reconstruction method is called demosaicing. This has long been a much-studied topic, especially for CFAs [117]. Demosaicing is based on spatio-spectral correlation [118,119,120], modelled either in the spatial domain or the frequency domain [121,122], by machine learning [123] or by any other non-linear methods [124,125]. Preliminary works have investigated jointly optimizing the sensor design with its associated demosaicing algorithm [126,127], but this has not yet been generalized enough in all dimensions.

A pixel has a response function that maps the portion of light collected by the image intensity *I* [128]. However, nonlinearities can be caused by the lack of sensor dynamic range, which is related to the ability to capture very weak light signals and very strong light signals simultaneously. This has been counterbalanced by exposure bracketing techniques [129,130,131], but makes instantaneous imaging impossible and prone to temporal artefacts [132]. For spectral cameras, the dynamic range of raw data can vary greatly from one channel to another, due to the variation in channel sensitivity responses [30,133]. One direction is to exploit these responses to emulate the exposure bracketing, i.e., to produce high dynamic range (HDR) images from a single raw image. We can call this method “exposure mosaicking”.

From a reconstructed spectral image, it is possible to obtain a pixel-by-pixel estimate of the spectral signature of a point in a scene. On most natural surfaces, spectra in the VNIR are known to be relatively smooth functions across wavelengths. Thus, a spectral signature can be estimated using a linear combination of spectral channels.

A set of known reflectance spectra is used to calibrate the system from intensity measurements ($I_{s}\left(X\right)$ in Equation (2)). Many other inverse problems have been solved using estimated spectral data, such as reflectance estimation (r(λ) in Equation (2)) [22,104,134,135,136], illuminant estimation (l(λ) in Equation (2)) [137,138], or camera characterization (s(λ) in Equation (2)) [139,140,141]. These estimators are generally designed and evaluated independently of the technology. A major problem exists, however: the estimation performance depends on the prior spatial reconstruction and can vary greatly from system to system. This is illustrated in Figure 2.

### 4.2. Generalization of Reconstruction Methods

The reconstruction of the different dimensions, as seen above, is usually designed and evaluated individually, algorithm after algorithm. However, the optimization of an imaging pipeline must consider the joint optimization of all blocks, whereas optimizing a block independently of the others remains only an academic curiosity, as Li et al. discussed [142]. Additionally, algorithms are often specific to the capture technology used, and their individual contribution to image quality is not evaluated within an overall pipeline. Therefore, there is a need to unify the imaging pipeline. We propose below two frameworks that would allow for the definition of an unified image reconstruction:

1. An approach could consider each block of the imaging pipeline as a parameterized box. Thus, it is possible to optimize all these parameters using an optimization process. An attempt of this kind has already been proposed in the literature [83], but with limited generalization purposes.
2. Another approach is to have one single optimized block that encapsulates the whole imaging pipeline, where effective parameters may not have any meaningful semantic meaning. This set of parameters could be determined by an optimizer that takes raw spectral data and their metadata as input and provides a full-resolution HDR hyperspectral image. This is what is illustrated in Figure 4.

For both approaches, linear and non-linear methods can be considered, and the optimization can be carried out over several SSI technologies and several datasets. In any case, these approaches require a quality measure (objective cost functions), with quality coverage across all dimensions.

### 4.3. Spectral Imaging and Polarization

A branch of unconventional imaging is polarization imaging. In general, this polarization signature is measured independently at a specific wavelength. When considered in conjunction with spectral imaging, this is often because the polarization signal is a noise effect [143]. However, polarization imaging has recently made it possible to estimate scene characteristics in the RGB domain, such as the illumination colour/direction [144,145], the surface normals of an object [146], or the diffuse/specular components [147]. Some researchers have already designed algorithms that exploit the correlation between spectral and polarization data for image reconstruction [148,149]. Nowadays, new cameras enable the instant capture of linear polarization information, first in a specific spectral band [150] and then in RGB (IMX250 MZR/MYR commercial sensors [151]). Although academic prototypes exist [152], it is conceivable that, in the near future, instantaneous multispectral polarization imaging systems will be available on the market. Thus, it will be important to take into account polarization sensitivities in instantaneous spectral systems, either by exploiting this modality for a better scene characterization, or for image restoration (e.g., removing highlights).

Most of the work on spectro-polarimetric imaging has been conducted in the visible wavelength range. Microfiltering technologies also extend to polarization optics and enable the fabrication of a polarization-sensitive sensor in other wavelength bands such as SWIR, MWIR, or thermal bands [153,154]. It is necessary to develop new spectro-polarimetric models and usages, investigate characterization/calibration techniques, and adapt computational imaging methods to these specific wavelengths.

Finally, spectral routers or microfilters may exhibit slight polarization dependence. Indeed, the materials used to manufacture the optical components of the spectral router (e.g., waveguides) may have birefringent properties, which can induce polarization dependence. It would be relevant to characterize this dependence, with a view to using the polarization information for various purposes mentioned above.

### 4.4. Spectral Video and Green Media

Time series are sequences of data points indexed by time, typically obtained by observing a random variable over consistent intervals. Spectral image reconstruction can take advantage of the temporal modality when a spectral video is available (a sequence of spectral images). Conventionally, solving a problem with deep learning involves initializing a neural network architecture randomly and fitting it with the training data. However, when the training dataset is limited, which is often the case when using hyperspectral images [155], time series can lead to overfitting, where the model adapts too closely to the training data, resulting in poor performance. There is still the need to define a strategy to exploit the temporal dimension of SSI images, by adapting time series tools to the specific case of spectral video data.

Spectral data is often large and complex to process and transmit. This is even more so the case for spectral videos. That is why spectral imaging will require dedicated system-on-chips and a hardware/software pipeline, also called image signal processing (ISP), to achieve ambitious timing constraints with low latency and jitter. This, together with the standardization discussed in Section 3, will enable the support of video bitrates and the development of real-time applications. It is now necessary to consider practical recommendations for obtaining good image quality with reduced computing time. What follows is a list of criteria that could be investigated: data type (via the fixed-point approximation of spectral data), the parallelization of processing tasks, dimensionality reduction, cache optimization, or the use of low-level languages. These practical considerations are closely related to energy consumption, and this is an even more relevant topic, since European policy promotes the ecodesign of products and has established minimum energy requirements for many products (e.g., Commission Regulation (EU) 2019/2021 of 1 October 2019).

## 5. Image Quality

Spectral imaging is used in a wide range of applications, including remote sensing, medical imaging, and cultural heritage, as well as their various sub-fields (e.g., remote sensing for glaciology, agriculture, etc.), each with distinct objectives and quality requirements. Consequently, the notion of image or video *quality* is generally ill-defined. For instance, in remote sensing, image quality may be defined in terms of cloud coverage and the prominence of shadows [156,157]. In medical imaging, quality is often tied to the clarity of diagnostically relevant features, such as the detection of tumours or vascular anomalies [158]. In cultural heritage, quality might relate to the accurate reproduction of colour and texture for the purpose of preservation, restoration, or visualisation [159]. In most cases, these assessments are influenced by factors such as the sensor’s spectral sensitivity, spatial resolution, signal-to-noise ratio, and calibration and reconstruction procedures. However, the relative importance of these factors shifts depending on the specific problem, leading to a fluid and application-specific understanding of what constitutes high-quality spectral data. This challenge is further compounded by the high dimensionality of spectral data, which introduces computational challenges [160]. Another enduring difficulty is obtaining robust and unbiased ground truth (GT) data, a challenge emphasised by Chehdi et al. [161], who noted that available GT datasets can distort the physical characteristics of spectral data, thereby skewing classification results. As a consequence of these challenges, and despite the growing importance of spectral imaging since the 1990s, the development of well-accepted, standardised metrics for spectral image/video quality (and more specifically for SSI systems) has been relatively slow.

Importantly, image/video quality assessment can be defined with respect to a human end-user or a machine/algorithm, typically a combination of both. The former approach involves attributes such as aesthetics, naturalness, and saliency [162], and is relevant mainly in the context of *visualising* the spectral data (via rendering [163] or colour-compositing [164]). For instance, in monitoring and conservation, true-colour rendering of the spectral data helps researchers to intuitively understand vegetation health, such as by identifying obvious land degradation or deforestation. On the other hand, false colour compositing enables the visualisation of, e.g., infrared wavelengths, and the display of useful features informative of plant and soil health, which would not come across in a true-colour render [164]. Either way, the quality of the render/composite is dependent on the quality of the spectral data. The subjective analysis is typically complemented by objective methods for, e.g., semantic segmentation, object recognition, or anomaly detection, and the performance of such methods is naturally also dependent on the quality of the spectral data (e.g., compression artefacts may hinder segmentation, anomaly detection, etc. [165]). Therefore, for this application and many others, both technical (machine/algorithm end-user) and subjective (human end-user) quality criteria are important. In spectral cross-media reproduction, such as spectral printing [166], the sole purpose of spectral data is to produce renders/prints (e.g., for different illuminants). In such an application, subjective quality criteria are the most relevant [167]. On the other hand, there are use cases where only technical quality matters, in the sense that the spectral data is never directly visualised. In such cases, a heat map or a segmentation map may be used as proxy to visualise the data (e.g., nutrient mapping in precision agriculture).

Simple quantitative measures, such as the root mean square error (RMSE), the goodness of fit coefficient (GFC) or the peak signal-to-noise ratio (PSNR), are well established for their simplicity and computational efficiency (see, e.g., [168]), but, because they are pixelwise metrics (i.e., not accounting for the spatial arrangement of pixels in the image/video), they typically fail to capture meaningful quality attributes. For instance, a one-pixel misalignment can yield a substantial RMSE, despite a potentially negligible impact on the interpretation, be it subjective or objective. Researchers have also adapted well-known perceptual indices, including the structural similarity index (SSIM), to account for spectral aspects [169,170,171,172]. In these works, e.g., MvSSIM [172], the lack of information on spectral structures carried by an average of SSIM computed on each spectral bands are exposed, and proposals to overcome this limitation have been developed. This approach is promising but has yet to be confirmed useful in different application domains, especially for technical quality assessment. Research in greyscale and colour image quality assessment has inspired other proposals for dedicated spectral quality metrics, including via the use of scene statistics and machine learning [173,174,175,176,177], although these models can be difficult to interpret and computationally demanding (which also raises environmental concerns). Furthermore, the question of the usefulness and transferability of these models across application domains remains open.

A possible direction is a multi-dimensional “quality space” or “quality gamut” that goes beyond single-metric evaluations. It would cover fundamental dimensions such as resolution, signal-to-noise ratio, and radiometric accuracy and would include domain-specific requirements. This would contribute to unifying and standardising methodological best practices, bridging the gap between narrow, application-specific demands and the broader call for transparent, consistent metrics in spectral imaging. A potential direction for such approach is introduced in [178], for visual colour content, where the different quality attributes of printed colour images are identified. It is, however, necessary to quantify them by the definition of specific indicators and by the quantification of their weights on the image quality.

## 6. Conclusions

We have surveyed the different aspects of SSI and emphasized different promising future research directions. We propose considering the system as a whole, assuming it is parametrized by specific features that may or may not be semantically meaningful; if this is supported by adequate quality evaluation and standardized procedures, this should greatly improve SSI systems. If we recall the example in Figure 2, then the two estimated spectral information from two different systems would have a better quality and a traceable uncertainty, which would allow for better interpretation and communication.

At the fundamental level, it appears important to provide an environment and a method to design and study spectral imaging. This could be supported by the unification of the quality indices, the unification of the image processing workflow, and the unification of the system qualification, i.e., standards and calibration.

At the realisation level, it seems possible to develop new SSI systems that outperform those in the the current literature. This is made possible by both the freedom and efficiency in the design of filters enabled by new manufacturing techniques, together with fundamental advances in sensor design.

At the application level, SSI has the potential to allow new discoveries in different scientific fields, thanks to the time series analysis of processes that could not be observed before. Significant advances in imaging could become possible through widespread access to and use of new generations of spectral imaging systems that enable the observation and characterization of rapid dynamic processes.

SSI could be deployed in society; however, current solutions are not sufficient to ensure the validity and performance of systems, as users still need to access a formal framework for the use of SSI. This is particularly true for the following aspects: standardization and ecodesigns must be developed, so that the amount of data to be processed, stored, and transmitted decreases. Potential directions can be taken depending on the hardware, such as the potential parallelization of tasks, the optimization of data types, or dimensionality reduction. This could help meet the real-time temporal constraints needed for future spectral video systems.

## Data Availability

Data are contained within the article.
